# Some American Hospitals

**Published:** 1892-01-30

**Authors:** 


### Some American Hospitals.

By Our Special Commissioner.

### V.—MILITARY HOSPITALS.

We had an opportunity, thanks to the courtesy of Dr. Perley, Dr. O'Reiley, and Dr. Wolferton, of inspecting more than one military hospital during our progress through the States.

It is pleasing to note the professional attainments and scientific knowledge displayed by the surgeons attached to the Surgeon-General's Department of the United States army. Any Englishman interested in hospitals visiting America should take care to avail himself of the opportunity of being in the neighbourhood of one of the forts where troops are stationed to pay a visit to the surgeon in charge, as he is sure to meet with a most courteous reception, and to have a delightful time in many ways.

There is a fairly large military hospital at Presidio, San Francisco, which we did not go over, and a smaller one at Fort Mason. This latter institution is in charge of Dr. Perley, and it was pleasing to note the very high state of efficiency to which he had brought it. Every detail pointed to his thoughtful and intelligent care, sound knowledge, and efficient management. We can heartily congratulate Dr. Perley upon the state of his hospital in which he takes a legitimate pride, and we can only wish that many of the American hospitals were anything like as efficient as that of the military one at Fort Mason.

We also paid a visit to Fort Logan, near Denver, in charge of Surgeon O'Reilley. This hospital is on the pavilion plan, being of the straight type. It struck us during our visit, as we had also noticed elsewhere, that the American Government seems to spare neither expense nor trouble in making its soldiers thoroughly comfortable. Not only is this the case with regard to the hospitals, but every detail of the barrack arrangements of these well-managed forts proved that the lot of a soldier of the United States army is probably better than that of any soldier in any other army throughout the civilised world.

The hospital at Fort Logan consists of two wards, of eighteen beds each, on the ground floor, with a kitchen and dining-room behind, it being the custom that all convalescent patients shall take their meals outside the wards whenever possible. Upstairs are two wards, similar in all respects to those on the ground floor, and also rooms for the attendants.

Each of these military hospitals contains a good library, well supplied with periodical and other medical literature, at the expense of the United States Government.

We do not know how far this fact may be responsible for the circumstance that the medical officers in charge of the military hospitals throughout the United States display an intelligence and scientific knowledge rarely to be met with in members of the profession serving with troops in other countries. The work, so far from causing them to lose their interest in their profession, the surgeons in the Surgeon-General's Department of the United States army seem to acquire a scientific intelligence that makes them, so far as our experience goes, remarkable for their knowledge and attainments.

It may interest many people to learn that the cooks in charge of the military hospitals take a very great pride in providing the best of food in the most appetising manner in order to tempt the appetites of the patients. By the courtesy of Dr. O'Reiley we are able to give the following menu, supplied by the cook of the hospital at Fort Logan, on September 21st, 1891:—Breakfast: Beef hash, mince pie, Washington pie, coffee, bread, and butter. Dinner: Roast beef, gravy, mashed potatoes, tomatos, common starch pudding, French sauce, pumpkin pie, coffee, and bread. Supper, Beef pie, apple pie, Spanish cream, sponge jellycake, bread: butter, &c. It is very likely that many of the dishes will be unknown to English readers, but they may take it from us that everyone of them is not only appetising but excellent.

